# The Global Health System: Actors, Norms, and Expectations in Transition

**DOI:** 10.1371/journal.pmed.1000183

**Published:** 2010-01-05

**Authors:** Nicole A. Szlezák, Barry R. Bloom, Dean T. Jamison, Gerald T. Keusch, Catherine M. Michaud, Suerie Moon, William C. Clark

**Affiliations:** 1 Sustainability Science Program, John F. Kennedy School of Government, Harvard University, Cambridge, Massachusetts, United States of America; 2 Harvard School of Public Health, Boston, Massachusetts, United States of America; 3 Department of Global Health, University of Washington, Seattle, Washington, United States of America; 4 Global Health Initiative, Boston University, Boston, Massachusetts, United States of America; 5 Harvard Initiative for Global Health, Harvard University, Cambridge, Massachusetts, United States of America; London School of Hygiene and Tropical Medicine, United Kingdom

## Abstract

In the first in a series of four articles highlighting the changing nature of global health institutions, Nicole Szlezák and colleagues outline the origin and aim of the series.

*This is the first in a series of four articles that highlight the changing nature of global health institutions.*

## The Global Health System: A Time of Transition

The global health system that evolved through the latter half of the 20^th^ century achieved extraordinary success in controlling infectious diseases and reducing child mortality. Life expectancy in low- and middle-income countries increased at a rate of about 5 years every decade for the past 40 years [1]. Today, however, that system is in a state of profound transition. The need has rarely been greater to rethink how we endeavor to meet global health needs.

We present here a series of four papers on one dimension of the global health transition: its changing institutional arrangements. We define institutional arrangements broadly to include both the actors (individuals and/or organizations) that exert influence in global health and the norms and expectations that govern the relationships among them (see Box 1 for definitions of the terms used in this article).

**Box 1. Defining the Global Health System**

We understand global health needs to include disease prevention, quality care, equitable access, and the provision of health security for all people [16]–[18]. We define the global health *system* as the constellation of actors (individuals and/or organizations) “whose primary purpose is to promote, restore or maintain health” [19], and “the persistent and connected sets of rules (formal or informal), that prescribe behavioral roles, constrain activity, and shape expectations” [20] among them. Such actors may operate at the community, national, or global levels, and may include governmental, intergovernmental, private for-profit, and/or not-for-profit entities.

The traditional actors on the global health stage—most notably national health ministries and the World Health Organization (WHO)—are now being joined (and sometimes challenged) by an ever-greater variety of civil society and nongovernmental organizations, private firms, and private philanthropists. In addition, there is an ever-growing presence in the global health policy arena of low- and middle-income countries, such as Kenya, Mexico, Brazil, China, India, Thailand, and South Africa.

**Figure 1:**
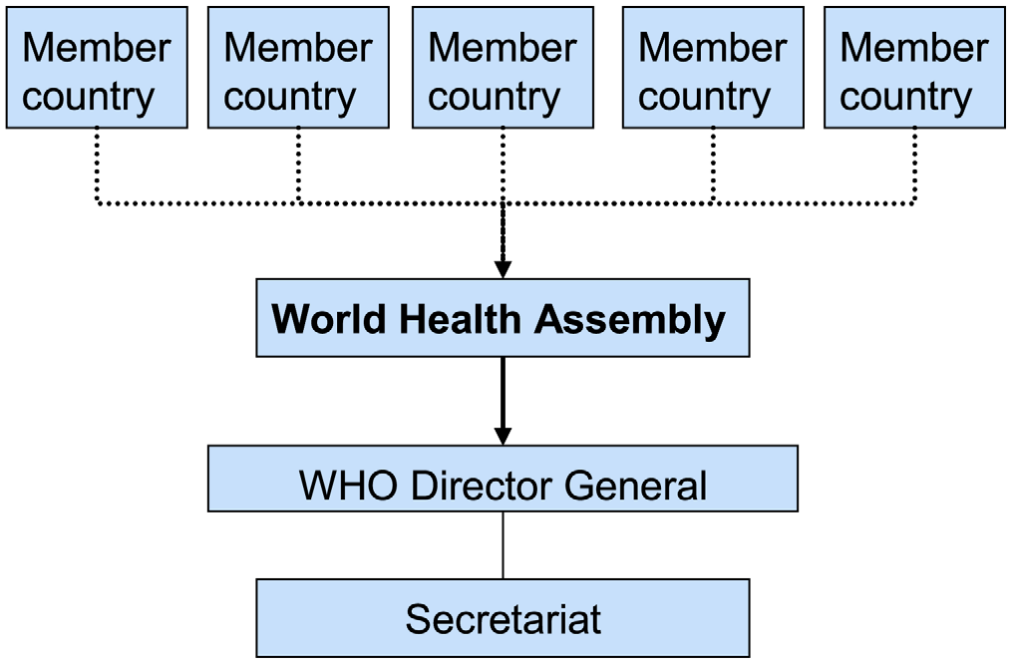
UN-type international health governance. Based on the principles of the UN system, member countries are represented in the World Health Assembly (WHA), which functions as the central governing body. The WHA appoints the director general, oversees all major organizational decision making and approves the program budget.

**Figure 2:**
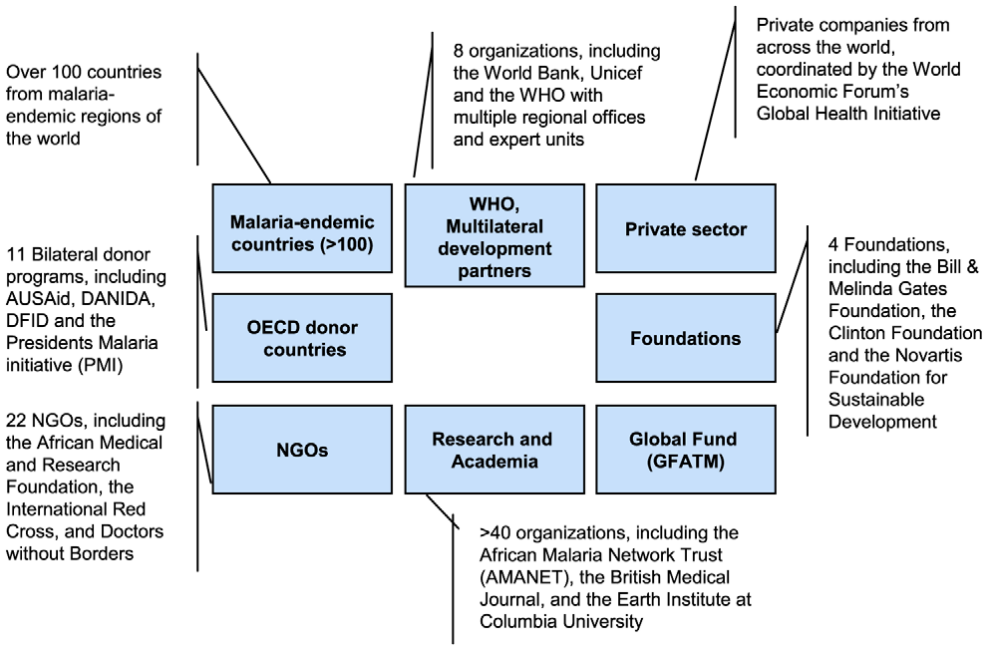
Global Health as partnership. Today's Roll Back Malaria Partnership consists of more than 500 partners, including the major players WHO, the Global Fund, and the Bill & Melinda Gates Foundation. RBM was initiated in 1998 by WHO, UNICEF, UNDP, and the World Bank. WHO currently hosts RBM's secretariat and contributes in multiple ways. However, it is not presented as the central node of the partnership (source: http://www.rollbackmalaria.org/).

Also changing are the relationships among those old and new actors—the norms, expectations, and formal and informal rules that order their interactions. New “partnerships” such as WHO's Roll Back Malaria Partnership (RBM), Stop TB, the Global Alliance for Vaccines and Immunization (GAVI), the Global Fund to Fight AIDS, Tuberculosis and Malaria (GFATM), and many others have come to exist alongside and somewhat independently of traditional intergovernmental arrangements between sovereign states and UN bodies (see Figures 1 and 2 for an illustration of the underlying governance principles). These partnerships have been emphasized—not least by WHO itself—as the most promising form of collective action in a globalizing world [2]. Large increases in international support for the newer institutions has led to relative and, in some cases, absolute declines in the financial importance of traditional actors [3].

The rise of multiple new actors in the system creates challenges for coordination but, more fundamentally, raises tightly linked questions about the roles various organizations should play, the rules by which they play, and who sets those rules. Actors may exercise power within the constraints of international institutions in hopes of achieving benefits and shared objectives [4]. Such a calculus helps to explain why actors are willing to fund multilateral initiatives such as WHO, GFATM, RBM, and Stop TB, despite the fact that doing so entails relinquishing considerable control over what is done with their resources. On the other hand, powerful and financially independent actors, such as national governments, may elect to use their resources to influence the outcomes from multilateral initiatives or create bilateral ones. The lack of a clear set of rules that constrain distortion of priorities by powerful actors can threaten less powerful ones. As a case in point, despite widespread support for its overarching goals, there is considerable discussion, in some cases even unease and some tension, around the prominent role played by the Bill & Melinda Gates Foundation, whose spending on global health was almost equal to the annual budget of WHO in 2007 [5]–[8].

Finally, this period of transition in actors and relationships comes at a time when the very nature of the challenges faced by health systems is itself being transformed. The success of child survival efforts has meant that noncommunicable diseases, including cardiovascular disease, cancer, diabetes, and neuropsychiatric disease, are growing in prevalence alongside the continuing threats of communicable diseases [9]–[11]. The globalizing economy poses a new set of health challenges as the rules that govern trade in goods, services, and investment reach more deeply into national regulatory and health systems than have previous trade arrangements [12],[13]. Finally, changes in climate and other environmental variables are likely to create unexpected and unpredictable health threats, both as a direct result of changing environments for disease vectors and as an indirect result of impacts on water and food security, extreme events, and increased migration [14],[15].

The melee resulting from these interacting transitions has produced some extraordinary success stories, such as the drive that dramatically increased access to lifesaving antiretroviral therapy for people living with HIV/AIDS, unprecedented access to insecticide-treated bednets for malaria, and enhanced access to anti-TB drugs in the developing world within a span of a few short years. But there is also mounting concern that the increasingly complex nature of the evolving global health system leaves unexploited significant opportunities for improving global health, results in duplication and waste of scarce health resources, and carries high transaction costs. The ongoing global financial crisis makes the efficient and effective performance of the global health system all the more pressing.

Many have expressed doubts that today's global health system is remotely adequate for meeting the emerging challenges of the 21^st^ century [21]–[24]. A groundswell of opinion [25]–[35] suggests that new thinking is needed on whether or how practical reform of the present complex global health system can improve its ability to deal with such key issues as:

- Setting global health agendas in ways that not only build upon the enthusiasm of particular actors, but also improve the coordination necessary to avoid waste, inefficiency, and turf wars.
- Ensuring a stable and adequate flow of resources for global health, while safeguarding the political mobilization that generates issue-specific funding. How can the global burden of financing be equitably shared, and who decides? How should resources be allocated to meet the greatest health risks, particularly those that lack vocal advocates?
- Ensuring sufficient long-term investment in health research and development (R&D). Who should contribute, and who should pay? How can the dynamism and capacity of both public and private sectors from North and South be harnessed, without compromising the public sector's regulatory responsibilities?
- Creating mechanisms for monitoring and evaluation and judging best practices—how can policy agreement be achieved when actors bring contested views of the facts to the table?
- Learning lessons from the enormous variance in effectiveness and costs of various national and international health systems, from R&D to the delivery and monitoring and evaluation (M&E) of interventions in the field, to create improvements everywhere.

## Roadmap of the Series

In this series we undertook a study of the role of institutions in the global health system. The aims of the study were threefold: first, to advance current understanding of the interplay of actors in the system; second, to evaluate its performance; and third, to identify opportunities for improvement. The project was part of a larger program led by Harvard University's John F. Kennedy School of Government to advance thinking on the challenges of linking research knowledge with timely and effective action in an increasingly globalized and diverse world [36],[37]. It drew together theoretical literature on global governance that has emerged from the field of international relations over the last half-century [20],[38],[39]; on empirical analysis of institutional design and performance in other sectors that, similar to public health, seek to mobilize scientific knowledge as a global public good (e.g., agriculture and environmental protection [40]–[42]); and on the engagement of several of the authors of this paper in contemporary policy debates on ways to improve the institutions that promote global health [43],[44].

We focused on three central questions regarding the global health system: (1) What functions must an effective global health system accomplish? (2) What kind of arrangements can better govern the growing and diverse set of actors in the system to ensure that those functions are performed? (3) What lessons can be extracted from analysis of historical experience with malaria to inform future efforts to address them and the coming wave of new health challenges? To illuminate these questions, we built a series of case studies, workshops, and synthesis efforts, the results of which are reported in more detail elsewhere (http://www.cid.harvard.edu/sustsci/events/workshops/08institutions/index.html).

In the papers presented in this series we summarize representative results from our work for one key actor in, and one key function of, the global health system. Thus, the second article in the series, by Frenk [45], reflects on the essential characteristics of functioning national health systems, which are the anchoring institutions of the global health system. The continued crucial importance of national health systems as connectors of research and development with populations, and as guarantors of the successful and sustained delivery of health interventions to people and populations, is often overlooked in enthusiastic discussions of new approaches to the architecture of global health. Indeed, the biggest challenge facing global health today is to reconcile the ongoing global-level transformation with the need to further strengthen and support national-level health systems.

The third article, by Keusch et al. [46], examines how the global health system has evolved to better integrate the research, development, and delivery of health interventions—a core function of the system. We chose the global response to malaria as a good case study because of the long history of global efforts to combat the disease, multiple attempts at institution building in this domain, its recent rise on the global agenda, and the concomitant increase in resources devoted to combating it. Many old and new approaches have evolved and been tested in the field of malaria, including targeted programs like WHO's Malaria Action Programme and the WHO/UNDP/Unicef/World Bank Training in Tropical Diseases (TDR) Programme; governance partnerships like RBM; product development partnerships such as the Medicines for Malaria Venture; and new delivery mechanisms such as GFATM. Goals have oscillated between global eradication, regional and national control, and now perhaps back to global eradication. Exploration of the evolution of institutional arrangements linking malaria research, development, and delivery hold important lessons for understanding the global health system more generally.

The fourth article of the series, by Moon et al. [47], presents conclusions regarding the three central questions raised above and poses questions for further research and recommendations for future action.

Our hope is that this series stimulates debate, encourages further case studies, and provides insights into general principles for the improvement of the global health system.

## Abbreviations

GAVI: Global Alliance for Vaccines and Immunization
GFATM: Global Fund to Fight AIDS, Tuberculosis and Malaria
M&E: monitoring and evaluation
R&D: research and development
RBM: Roll Back Malaria Partnership
WHA: World Health Assembly
WHO: World Health Organization
